# Vasopressin and terlipressin in adult vasodilatory shock: a systematic review and meta-analysis of nine randomized controlled trials

**DOI:** 10.1186/cc11469

**Published:** 2012-08-14

**Authors:** Ary Serpa Neto, Antônio P Nassar, Sérgio O Cardoso, José A Manetta, Victor GM Pereira, Daniel C Espósito, Maria CT Damasceno, James A Russell

**Affiliations:** 1 Medical Intensive Care Unit, ABC Medical School (FMABC), Lauro Gomes Avenue 1000, Santo André 09060-650, Brazil; 2 Department of Intensive Care Medicine, São Camilo Hospital, Pompeia Avenue 1178, São Paulo 05024-000, Brazil; 3 Heart and Lung Institute, Division of Critical Care Medicine, St Paul's Hospital, 1081 Burrard Street, Vancouver, BC V6Z 1Y6, Canada

## Abstract

**Introduction:**

Catecholamines are the most used vasopressors in vasodilatory shock. However, the development of adrenergic hyposensitivity and the subsequent loss of catecholamine pressor activity necessitate the search for other options. Our aim was to evaluate the effects of vasopressin and its analog terlipressin compared with catecholamine infusion alone in vasodilatory shock.

**Methods:**

A systematic review and meta-analysis of publications between 1966 and 2011 was performed. The Medline and CENTRAL databases were searched for studies on vasopressin and terlipressin in critically ill patients. The meta-analysis was limited to randomized controlled trials evaluating the use of vasopressin and/or terlipressin compared with catecholamine in adult patients with vasodilatory shock. The assessed outcomes were: overall survival, changes in the hemodynamic and biochemical variables, a decrease of catecholamine requirements, and adverse events.

**Results:**

Nine trials covering 998 participants were included. A meta-analysis using a fixed-effect model showed a reduction in norepinephrine requirement among patients receiving terlipressin or vasopressin infusion compared with control (standardized mean difference, -1.58 (95% confidence interval, -1.73 to -1.44); *P *< 0.0001). Overall, vasopressin and terlipressin, as compared with norepinephrine, reduced mortality (relative risk (RR), 0.87 (0.77 to 0.99); *P *= 0.04). Vasopressin compared with norepinephrine decreased mortality in adult patients (RR, 0.87 (0.76 to 1.00); *P *= 0.05) and in patients with septic shock (42.5% vs. 49.2%, respectively; RR, 0.87 (0.75 to 1.00); *P *= 0.05; number needed to treat, 1 to 15). There was no difference in adverse events between the vasopressin and control groups (RR, 0.98 (0.65 to 1.47); *P *= 0.92).

**Conclusions:**

Vasopressin use in vasodilatory shock is safe, associated with reduced mortality, and facilitates weaning of catecholamines. In patients with septic shock, use of vasopressin compared with norepinephrine may also decrease mortality.

## Introduction

The mortality rate of patients with shock remains high [1]. Vasodilatory shock is characterized by low arterial blood pressure due to a significantly decreased systemic vascular resistance. The most frequent causes of this type of shock are sepsis and post-cardiovascular surgery requiring cardiopulmonary bypass. However, massive vasodilatation can result from shock of any origin [1].

Aggressive volume resuscitation is the mainstay of initial shock management, followed by vasoactive infusions when fluids do not restore adequate arterial pressure and tissue perfusion [2]. Currently, catecholamines are the preferred vasopressor but the development of adrenergic hyposensitivity with the loss of pressor responsiveness makes finding other options necessary [3]. Additionally, catecholamines - especially dopamine and epinephrine - have significant adverse effects, such as decreased cardiac output and oxygen delivery, arrhythmia, and organ ischemia, especially at high doses. Catecholamines may even increase mortality rates [3].

Vasopressin is a neurohypophyseal hormone with diverse actions mediated by tissue-specific receptors. Low-dose vasopressin and its analog terlipressin have emerged as promising therapies in vasodilatory shock for several reasons. The rationale for using vasopressin and its analogs is the development of relative vasopressin deficiency in patients with vasodilatory shock and the observation that exogenously administered vasopressin restores vascular tone, increases responsiveness to infused catecholamines and raises blood pressure, thereby reducing the need for catecholamine use [4].

Observational and randomized controlled studies involving the use of vasopressin infusion in patients with vasodilatory shock have produced conflicting results. Our aim was to summarize these studies using a systematic review of the literature and a meta-analysis of randomized controlled trials focused on vasopressin and its analog terlipressin in adult patients with vasodilatory shock. We also evaluated vasopressin and terlipressin in studies of septic shock only.

## Materials and methods

### Search methods for identification of studies

Studies were identified using the Medline (1966 to 2011) and CENTRAL (1800 to 2011) databases using a sensitive search strategy combining medical subject headings and keywords (see Additional file 1 for details). Abstracts from recent major conferences (American Thoracic Society, European Society of Intensive Care Medicine, and Society of Critical Care Medicine) were searched for additional relevant studies. All of the review articles and cross-referenced studies from the retrieved articles were screened for pertinent information.

### Selection of studies

This meta-analysis was limited to studies that dealt with the role of vasopressin and/or terlipressin compared with catecholamine infusion in the treatment of vasodilatory shock in adult critically ill patients. Vasodilatory shock was defined as hypotension due to peripheral vasodilatation as result of failure of the vascular smooth muscle to constrict [2]. We included trials that satisfied the following inclusion criteria: randomized controlled trials comparing adult critically ill patients who had vasodilatory shock receiving treatment with vasopressin or terlipressin compared with patients not receiving such treatment; survival, biochemical and hemodynamic data; and patients in the studies receiving vasoactive infusion in the control arm and vasopressin plus vasoactive infusion in the experimental arm. Studies were excluded if survival outcome or biochemical and/or hemodynamic data were not provided, if pediatric patients were analyzed, or if they included patients without vasodilatory shock. When we found duplicate reports of the same study in preliminary abstracts and articles, we analyzed data from the most complete dataset.

### Data extraction

Data were independently extracted from each report by three authors, using a data-recording form developed for this purpose. After extraction, data were reviewed and compared by ASN. Disagreements between the two extractors were solved by consensus among the investigators. Whenever needed, additional information concerning a specific study was obtained by directly questioning the principal investigator.

### Definition of endpoints

The primary endpoint was overall survival. The survival time was defined as the time from randomization until death from any cause, or was censored on the date of the last follow-up assessment. Secondary endpoints included: change in hemodynamic variables; change in biochemical variables; decrease of catecholamine requirements; and assessment of adverse events.

### Statistical analysis

The effects of vasopressin and terlipressin on vasodilatory shock outcomes and the adverse effects of these drugs were examined. We extracted data regarding the study design, patient characteristics, treatment duration, medication, dosage, mean change for hemodynamic and biochemical variables, overall survival, and decreased catecholamine requirement. Changes in hemodynamic and biochemical variables were expressed as a percentage and were defined as the final value (after follow-up) minus the baseline value divided by the baseline value. The decreased catecholamine requirement was defined as any sustained decrease in catecholamine infusion over the study follow-up period. Norepinephrine and dopamine infusions were reported as micrograms per minute, vasopressin infusion was reported as units per minute, and terlipressin infusion was reported as micrograms per hour. The conversion of weight-based variables (μ/kg/minute) to time-based variables (μg/minute or μg/hour) was made by multiplying the value by the mean weight, either provided by the author or estimated as 70 kg. The conversion of bolus administration (mg) to continuous administration (μg/hour) was made by dividing the total daily dose by 24 and multiplying it by a conversion factor (mg to μg). All of the time variables were described as hours, and the other variables are described in the text.

For the survival analysis and for the proportion of patients who experienced adverse events, a pooled estimate of the relative risks (RRs) of the individual studies was computed using a fixed-effect model, according to Mantel and Haenszel, and these results graphically represented using forest plot graphs. For continuous variables, the standardized mean difference, which consists of the difference in means divided by the standard deviation, was used. The homogeneity assumption was checked by a chi-squared test with the degrees of freedom equal to the number of analyzed studies minus one. A sensitivity analysis was performed by recalculating the pooled RR estimates for different study subgroups based on the relevant clinical features. This analysis serves to show whether the overall result would be affected by a change in the meta-analysis selection criteria [5]. An estimate of the potential publication bias was carried out by plotting the single-study RR on a log scale against the respective standard error (funnel plot). For multiple comparisons, we used the Bonferroni correction method. Inter-rater reliability was determined by comparing the number of studies searched by Author 1 versus the number searched by Author 2 in each stage of the search by the kappa coefficient [5].

All variables were tested for normality using the Kolgomorov-Smirnov test. The parametric variables were described as the means and standard deviations, and the nonparametric variables were described as the medians and interquartile ranges. All of the analyses were made using Review Manager version 5.1 (Copenhagen: The Nordic Cochrane Center, The Cochrane Collaboration, 2011) and Statistical Package for the Social Sciences version 16.0 (SPSS Inc., Chicago IL, USA). For all analyses, *P *< 0.05 was considered significant. For publication bias, *P *< 0.1 was considered significant.

## Results

### Literature search

**Figure 1:**
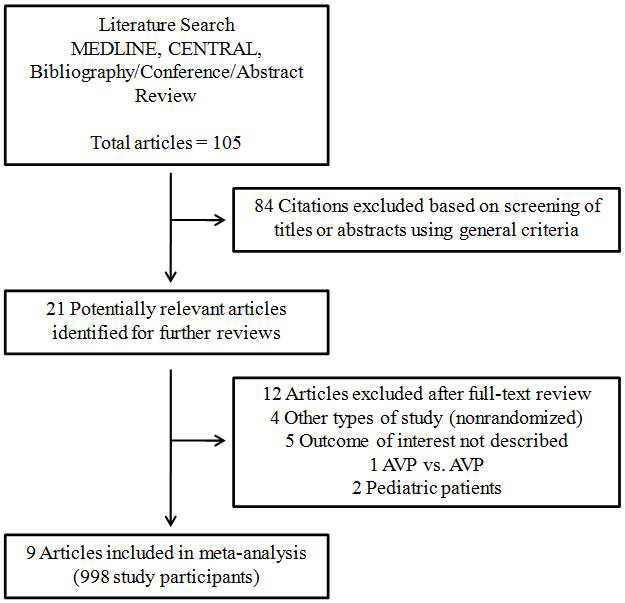
**Literature search strategy**. AVP, vasopressin.

The search strategy retrieved 105 unique citations. Of these citations, 84 were excluded after the first screening based on the abstracts or titles, leaving 21 articles for a full-text review (Figure 1). In this review, 12 articles were excluded for the following reasons: outcome of interest not described (*n *= 5); study design not appropriate (*n *= 4); pediatric patients (*n *= 2); and evaluated vasopressin in both arms (*n *= 1). Finally, nine articles (998 participants) were included in the meta-analysis [6-14]. For all of the comparisons of inter-rater reliability in each stage of the search, the kappa coefficient ranged from 0.87 to 0.93.

### Study characteristics

The characteristics of the nine selected studies are shown in Table 1. Six studies evaluated vasopressin as a therapeutic approach, two evaluated terlipressin and one study evaluated both. In seven studies the disease responsible for vasodilatory shock was septic shock exclusively, in one study the shock occurred after post-left ventricular assist device, and in the last study the shock occurred post cardiotomy. The mean dose of the drugs across the studies was 38.72 ± 40.14 μg/minute for norepinephrine, 0.055 ± 0.027 U/minute for vasopressin, and 59.03 ± 47.59 μg/hour for terlipressin. The mean age was 61.91 ± 6.29 in entire group, and the median follow-up time was 24 (4.00 to 48.0) hours. The assessment of the study quality is exposed in Table S1 in Additional file 2.

**Table 1:** Characteristics of the studies included in the systematic review

Study	Arms	*n*	Age (years)	Design	Disease	Dosage	Prognostic index	Time (hours)	MAP objective (mmHg)
Albanèse and colleagues [6]	N	10	65	RCT	Septic shock	119 μg/minute	29 (APACHE II)	6	65 ± 10
	TP	10	66	OL		50 μg/hour	28 (APACHE II)	6	
Dünser and colleagues [7]	N	24	68	RCT	Vasodilatory shock	58.8 μg/minute	49.7 (SAPS II)	48	>70
	N+AV	24	68	OL	(PS in 40%)	0.06 U/minute	51.6 (SAPS II)	48	
Morelli and colleagues [8]	N	15	64	RCT	Septic shock	15 μg/minute	58 (SAPS II)	48	70 ± 5
	N+TP	15	67	OL		110.5 μg/hour	62 (SAPS II)	48	
	N+AV	15	66			0.03 U/min	60 (SAPS II)	48	
Morelli and colleagues [9]	N	20	67	RCT	Septic shock	84 μg/minute	59 (SAPS II)	4	70 ± 5
	N+TP	19	66	OL	(N >0.9 μg/kg/minute)	16.6 μg/hour	60 (SAPS II)	4	
Russell and colleagues [10]	N	382	62	RCT	Septic shock	15 μg/minute	27.1 (APACHE II)	672	65 ± 10
	N+AV	397	59	DB	(N >5 μg/minute)	0.03 U/minute	27 (APACHE II)	672	
Argenziano and colleagues [11]	N+P	5	52	RCT	Vasodilatory shock	19.7 μg/minute	-	0.25	>70
	N+AV	5	52	DB	Post-LVAD	0.1 U/minute		0.25	
Patel and colleagues [12]	N	11	68	RCT	Septic shock	17 μg/minute	24 (APACHE II)	4	Physician decision
	N+AV	13	68	DB	(high doses of D)	0.06 U/minute	22 (APACHE II)	4	
Malay and colleagues [13]	N+P	5	56	RCT	Septic shock	12 μg/minute	26 (APACHE II)	24	>70
	N+AV	5	53	DB	0.04 U/minute		27 (APACHE II)	24	
Lauzier and colleagues [14]	N	10	58	RCT OL	Septic shock	28.1 μg/minute	23.5 (APACHE II)	48	>70
	N+AV	13	51		(<12 hours of shock)	0.09 U/minute	22. (APACHE II)	48	
Total	C	482	62.23 ± 5.70	-	-	38.72 ± 40.14	-	24 (4 to 48)	
	AV	472	59.60 ± 7.68			0.05 ± 0.02		36 (24 to 48)	-
	TP	44	66.33 ± 0.57			59.03 ± 47.59		6 (4 to 48)	

Data presented as mean ± standard deviation or median (interquartile range). AV, arginine vasopressin; C, control; D, drug that the patients were already receiving at baseline; DB, double-blind; LVAD, left ventricular assist device; MAP, mean arterial pressure; N, norepinephrine; OL, open-label; P, placebo; PS, post-cardiotomy shock; RCT, randomized controlled trial; TP, terlipressin.

### Vasopressin and terlipressin in vasodilatory shock: hemodynamic and biochemical effects

Table S2 in Additional file 2 shows the changes in the hemodynamic variables of the vasopressin, terlipressin, and control patients in each study and in a combined analysis. Compared with the control group, the vasopressin group showed a significant reduction in heart rate (-12.8 ± 6.14% vs. -1.62 ± 6.86%; *P *= 0.023), and a nonsignificant increases in central venous pressure (+19.0 ± 1.41% vs. +10.66 ± 9.29%; *P *= 0.083), stroke volume index (+14.0 ± 8.54% vs. +1.00 ± 2.94%; *P *= 0.05), and left ventricular stroke work index (+65.00 ± 5.65% vs. 29.0 ± 26.54%; *P *= 0.064). When compared with terlipressin group, the vasopressin group showed a nonsignificant smaller reduction in cardiac index (-4.00 ± 9.25% vs. -16.33 ± 4.04%; *P *= 0.070). Compared with the control group, the terlipressin group showed a significant reduction in heart rate (-17.3 ± 7.09% vs. -1.62 ± 6.86%; *P *= 0.011) and oxygen delivery index (-16.0 ± 2.64% vs. -2.0 ± 6.28%; *P *= 0.036), and a borderline significant reduction in the oxygen consumption index (-11.33 ± 6.02% vs. -1.00 ± 5.65%; *P *= 0.071).

Table S3 in Additional file 2 shows the changes in the laboratorial variables of the vasopressin, terlipressin, and control patients in each study and in the combined analysis. There were no differences among the groups according to the variables evaluated. In the analyses of the standardized mean difference, we found a significant difference between the terlipressin and control groups in the cardiac index (-0.44 (-0.87 to -0.02); *P *= 0.04) and oxygen delivery index (-0.79 (-1.23 to -0.36); *P *= 0.0004), and a tendency toward a reduction in the gastric PaCO_2 _gap difference (-0.47 (-0.96 to 0.01); *P *= 0.06) (Figures S1 to S5 in Additional file 3).

**Figure 2:**
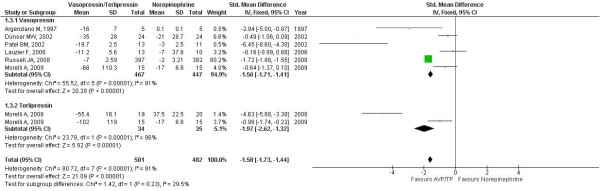
**Standardized mean difference for norepinephrine reduction between vasopressin/terlipressin and control**. Vasopressin (AVP) and terlipressin (TP) was associated with a significantly standardized mean difference with control (*P *< 0.0001 for both comparisons). CI, confidence interval; SD, standard deviation.

There was a significant reduction in the norepinephrine requirement among the patients receiving either a terlipressin or a vasopressin infusion. In the analyses of the standardized mean difference, there is a significant difference between the vasopressin and control patients (-1.56 (-1.71 to -1.41); *P *< 0.0001) and between the terlipressin and control patients (-1.97 (-2.62 to -1.32); *P *< 0.0001) with respect to the norepinephrine dosages (Figure 2).

### Vasopressin and terlipressin in vasodilatory shock: mortality and adverse events

**Figure 3:**
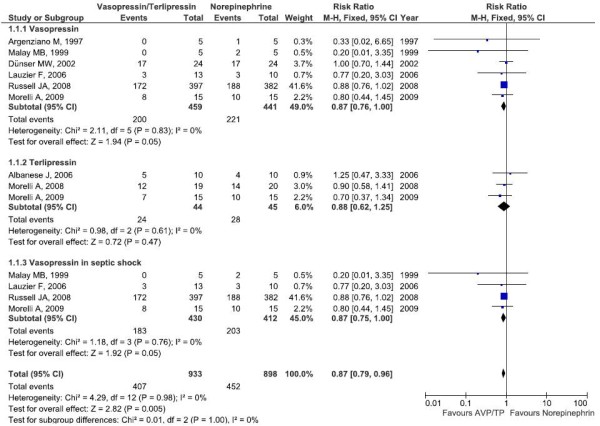
**Meta-analysis of overall survival for vasopressin, norepinephrine and terlipressin combined analyses**. Meta-analysis of overall survival for vasopressin (AVP) + norepinephrine (NE) or terlipressin (TP) + NE in vasodilatory shock, and for AVP + NE in septic shock. CI, confidence interval; M-H, Mantel and Haenszel.

The association of vasopressin with a decreased dose of norepinephrine infusion reduced mortality in adult patients (RR, 0.87 (0.76 to 1.00); *P *= 0.05). The terlipressin infusion did not influence mortality (RR, 0.88 (0.62 to 1.25); *P *= 0.47), and in the combined analysis terlipressin + vasopressin resulted in a decreased mortality (RR, 0.87 (0.77 to 0.99); *P *= 0.04) (Figure 3). With respect to adverse events, vasopressin and control patients were analyzed because no study that evaluated the effects of terlipressin described adverse events (see Table S4 in Additional file 2). There was no difference in adverse events between the vasopressin and control groups (RR, 0.98 (0.65 to 1.47); *P *= 0.92) (Figure S6 in Additional file 3). There is no correlation between the magnitude of the reduction of norepinephrine during follow-up and the risk ratio for mortality (*r *= 0.143; *P *= 0.652).

To explore the study heterogeneity, stratified analyses were performed across a number of key study characteristics and clinical factors. The summary of the analysis is shown in Table 2, and the complete analysis is shown in Table S5 in Additional file 2. In the survival analysis, we found a reduction in mortality with vasopressin patients with septic shock (*P *= 0.05) (Figure 3). The number needed to treat for this condition was 1 to 15. This effect on mortality disappeared after analysis without the study by Russell and colleagues [10]. The reduction in norepinephrine requirement was more significant in the double-blinded studies, in patients with septic shock, and with smaller doses of vasopressin and terlipressin. When we analyzed the data without the study by Russell and colleagues [10], the standardized mean difference found was smaller but still significant (Table S5 in Additional file 2).

**Table 2:** Summary of stratified analyses of pooled relative risks and standardized mean difference

Stratified analysis	Trials	*n*	Vasopressin	*P *value	Heterogeneity	Terlipressin	*P *value	Heterogeneity
**Mortality**								
Disease								
Septic shock	4	989	0.87 (0.75 to 1.00)	0.05	0.76	0.88 (0.62 to 1.25)	0.47	0.61
Nonseptic shock	2	58	0.95 (0.65 to 1.37)	0.77	0.46	-	-	-
**Norepinephrine reduction**								
Design								
Double-blind	3	813	-1.75 (-1.91 to -1.59)	<0.0001	<0.0001	-	-	-
Open-label	5	170	-0.45 (-0.85 to -0.06)	0.03	0.70	-1.97 (-2.62 to -1.32)	<0.0001	<0.0001
Disease								
Septic shock	6	925	-1.64 (-1.79 to -1.48)	<0.0001	<0.0001	-1.97 (-2.62 to -1.32)	<0.0001	<0.0001
Nonseptic shock	2	48	-0.66 (-1.22 to -0.11)	0.02	0.03	-	-	-
Vasopressin dosage								
≤0.05 U/minute	2	809	-1.66 (-1.83 to -1.50)	<0.0001	0.005	-	-	-
>0.05 U/minute	4	105	-0.77 (-1.22 to -0.32)	0.0008	<0.0001	-	-	-
Terlipressin dosage								
≤40 μg/hour	1	39	-	-	-	-4.63 (-5.88 to -3.38)	<0.0001	-
>40 μg/hour	1	30	-	-	-	-0.98 (-1.74 to -0.22)	0.01	-
**Cardiac index**								
Design								
Double-blind	3	46	0.94 (0.29 to 1.58)	0.004	0.45	-	-	-
Open-label	6	190	0.03 (-0.36 to 0.42)	0.89	0.80	-0.44 (-0.87 to -0.02)	0.04	0.54
Disease								
Septic shock	7	176	0.44 (-0.00 to 0.88)	0.05	0.10	-0.44 (-0.87 to -0.02)	0.04	0.54
Nonseptic shock	2	58	0.05 (-0.47 to 0.56)	0.86	0.70	-	-	-
Terlipressin dosage								
≤40 μg/hour	1	39	-	-	-	-0.29 (-0.92 to 0.35)	0.38	-
>40 μg/hour	2	50	-	-	-	-0.57 (-1.14 to -0.00)	0.05	0.37
Follow-up								
≤24 hours	5	103	0.94 (0.29 to 1.58)	0.004	0.45	-0.48 (-1.00 to 0.04)	0.07	0.28
>24 hours	4	131	0.03 (-0.36 to 0.42)	0.89	0.80	-0.37 (-1.09 to 0.35)	0.31	-
Patients								
≤25	5	87	0.52 (0.02 to 1.03)	0.04	0.12	-0.91 (-1.84 to 0.02)	0.06	-
25 to 50	4	147	0.08 (-0.36 to 0.53)	0.72	0.65	-0.32 (-0.80 to 0.15)	0.18	0.86
**Oxygen delivery**								
Terlipressin dosage								
≤40 μg/hour	1	39	-	-	-	-0.75 (-1.40 to -0.10)	0.02	-
>40 μg/hour	2	50	-	-	-	-0.83 (-1.42 to -0.25)	0.005	0.38
**Oxygen consumption**								
Terlipressin dosage								
≤40 μg/hour	1	39	-	-	-	-0.13 (-0.76 to 0.50)	0.69	-
>40 μg/hour	2	50	-	-	-	-0.52 (-1.08 to 0.05)	0.07	0.64
**Gastric PaCO_2 _gap**								
Vasopressin dosage								
≤0.05 U/minute	1	30	-0.38 (-1.11 to 0.34)	0.30	-	-	-	-
>0.05 U/minute	3	95	0.43 (0.01 to 0.84)	0.04	0.09	-	-	-
Terlipressin dosage								
≤40 μg/hour	1	39	-	-	-	-0.15 (-0.78 to 0.48)	0.64	-
>40 μg/hour	1	30	-	-	-	-0.94 (-1.70 to -0.18)	0.02	-

Data presented as relative risks (95% confidence interval) for mortality; and as the standardized mean difference (95% confidence interval for cardiac index, norepinephrine reduction, oxygen delivery, oxygen consumption, and gastric PaCO_2 _gap.

When analyzing only the double-blinded studies and the patients with septic shock, vasopressin and terlipressin were associated with an increase in cardiac index compared with norepinephrine alone. However, these findings were more significant in the studies with a shorter follow-up and a smaller number of patients. Higher doses of terlipressin (>40 μg/hour) were associated with decreases in cardiac index, oxygen delivery index, oxygen consumption index, and gastric PaCO_2 _gap difference. Further, higher doses of vasopressin (>0.05 U/minute) were associated with an increased gastric PaCO_2 _gap difference. Finally, bolus infusion of terlipressin was associated with a significant reduction of the oxygen delivery index and a borderline significant reduction of the cardiac index. These findings were not found with the continuous infusion. The Grading of Recommendations Assessment, Development and Evaluation (GRADE) evidence profile for the impact of vasopressin for the treatment of vasodilatory shock from this systematic review and meta-analysis of randomized controlled trials is shown in Table S6 in Additional file 2.

## Discussion

This systematic review suggests that the combination of vasopressin with norepinephrine in vasodilatory shock is safe, is associated with a reduction in patient mortality, and facilitates weaning of catecholamines, avoiding the latter's potential adverse events. Vasopressin and terlipressin did not decrease the cardiac or oxygen delivery indices. All of these changes are more significant in patients with septic shock. The stratified analysis of vasopressin combined with norepinephrine in patients with septic shock was associated with a borderline significant increase in hospital survival when compared with norepinephrine alone.

Vasodilatory shock pathogenesis is multifactorial. Increased nitric oxide, consequent to the activation of inducible nitric oxide synthase, is a major contributor to vasodilatation, acting both directly and via cyclic guanosine monophosphate to lower intracellular calcium levels, to decrease myosin light chain phosphorylation, and to activate calcium-sensitive (KCa) and adenosine triphosphate-sensitive (K_ATP_) K^+ ^channels [1,15]. An increasing number of studies have consistently found that patients who have vasodilatory shock and are evaluated in the ICU setting have very low plasma levels of vasopressin [16]. Hence, a relative deficiency of vasopressin may also be crucial to the altered functional status of vascular smooth muscle.

The clinical use of vasopressin has followed observations suggesting that exogenous administration of vasopressin during shock increases systemic blood pressure [17]. Blood pressure restoration does not necessarily improve outcome, however, if the increased blood pressure is accompanied by a worsening of cardiac performance and by decreased cardiac output and oxygen delivery [18,19]. High doses of vasopressin are associated with decreased cardiac output, oxygen delivery and consumption, and with increased gastric PaCO_2 _gap difference [20]. High-dose vasopressin is thus not indicated as an alternative to other vasopressors for the treatment of vasodilatory shock.

One important limitation to address is that the effects detected by our paper might be related to the decreasing doses of catecholamines instead of the fixed dose of additional vasopressor (although we did not find a relationship between reduction of norepinephrine and mortality). There is a current trend for decatecholaminization of patients, because catecholamines have deleterious effects on immune function, thrombogenicity and metabolic efficiency, on stimulating bacterial growth, and on causing myocardial injury [21]. In fact, higher mortality was noted in those patients where higher mean blood pressure values were generated using a progressively higher catecholamine dose [21].

We also found that vasopressin significantly reduced the heart rate in patients with vasodilatory shock without changes in cardiac output. This is an important finding because it could prevent the development and/or progression of the myocardial dysfunction associated with septic shock and the tachycardia-induced cardiomyopathy [22]. Recent studies also suggest that diastolic dysfunction is a common finding in patients with septic shock and is a major predictor of mortality in these patients [23]. As we already know, reducing the heart rate in patients with diastolic dysfunction can achieve adequate ventricular filling.

We found differences between the regimen used for administration of terlipressin (bolus versus continuous). Previous studies have suggested that intermittent bolus injection may be associated with significant adverse effects, including excessive microregional and systemic vasoconstriction, as well as decreases in cardiac output and oxygen delivery [24]. Conversely, recent studies provide evidence that continuous infusion of low-dose terlipressin exerts beneficial hemodynamic effects with reduced side effects as compared with traditional bolus injection. In general, continuous low-dose terlipressin was associated with improved parameters of myocardial contractility and renal function as well as less vascular leakage compared with bolus injection [24].

The largest randomized controlled trial of vasopressin infusion in septic shock showed no benefit of vasopressin versus norepinephrine [10]. However, this study has some interesting points to be discussed. First, it was found in the vasopressin-treated patients with less severe shock that the 25.8% relative reduction in 28-day mortality compared with norepinephrine was both striking and significant. This result is consistent with the evidence of a better synergistic effect of low-dose vasopressin in isolated arteries with conditions mimicking less severe septic shock [25]. Secondly, the dose of vasopressin chosen is lower than that generally used. Thirdly, the mortality in the control group is lower than the 60% anticipated in the sample size calculation, which could make the study underpowered to detect a significant difference in the outcome. Finally, the mean norepinephrine dose at randomization was considered too low by some studies [26]. However, in our stratified analysis no relationship between the baseline dose of norepinephrine and outcome was found (data not shown).

Polito and colleagues recently published a meta-analysis of vasopressin in vasodilatory shock with important differences from our study [27]. First, we analyzed only adult patients because pediatric shock has a much lower mortality than adult shock and this may contaminate the overall results. Also, we performed a more robust electronic search of references that resulted in the addiction of one study [11] that was missed by Polito and colleagues. Finally, we evaluated a higher number of variables, conducted an analysis based on the standardized mean difference of reduction in norepinephrine requirement, and performed a more robust sensitivity analysis.

These reported findings should be viewed within the context of the limitations of this study and research in the field. Although our literature search procedures were extensive, other trials may have appeared or may not have been published, and publication bias is therefore possible, which could overestimate the efficacy of these treatments. The assessment of adverse effects was limited to those studies in which adverse events were explicitly reported. The changes in hemodynamic and biochemical variables were calculated, and the standard deviations were probably biased because the correlation within patients is not reported. In addition, another limitation of our meta-analysis is that it is dominated by the largest randomized controlled trial from Russell and colleagues [10]. Finally, assumptions used to calculate the drug dosage may have influenced the results.

## Conclusions

The present meta-analysis has demonstrated the benefit of the association of vasopressin and terlipressin in reducing norepinephrine requirements in patients with vasodilatory shock, particularly in patients with septic shock. Our results show that vasopressin treatment is not associated with decreased cardiac output or oxygen delivery and consumption, even in higher doses. However, the pooled analyses showed that higher doses of terlipressin were associated with worsening of these variables. Vasopressin significantly reduces mortality in general patients, and specifically in patients with septic shock.

## Key messages

• Vasopressin and terlipressin reduce the norepinephrine requirements in patients with vasodilatory shock.

• Vasopressin is not associated with decreased cardiac output or oxygen delivery and consumption.

• Vasopressin significantly reduces mortality in patients with septic shock.

## Abbreviations

ICU: intensive care unit; PaCO_2_: partial pressure of carbon dioxide; RR: relative risk.

## Competing interests

JAR reports receiving consulting fees from Ferring Pharmaceuticals. There was no financial support for the manuscript. JAR reports holding stock in the University of British Columbia, which has submitted a patent related to the use of vasopressin in septic shock. JAR reports holding stock in Sirius Genomics Inc., which has submitted patents owned by the University of British Columbia and licensed to Sirius Genomics that are related to the genetics of sepsis and its treatment. JAR reports receiving consulting fees from Astra Zeneca, from BioCritica, and from Sirius Genomics Inc. JAR reports receiving grant support from Sirius Genomics, Ferring Pharmaceuticals, Astra Zeneca and Eli Lilly. JAR has received speaking honoraria from Pfizer and Eli Lilly. The remaining authors declare that they have no competing interests.

## Authors' contributions

ASN participated in the concept and design of the study, data acquisition, statistical analysis and interpretation, drafted the manuscript, and revised the manuscript for important intellectual content. APNJr participated in the data interpretation, drafted the manuscript, and revised the manuscript for important intellectual content. SOC participated in the data acquisition and interpretation, drafted the manuscript, and revised the manuscript for important intellectual content. JAM participated in the data interpretation, drafted the manuscript, and revised the manuscript for important intellectual content. VGMP participated in the data acquisition, and interpretation, drafted the manuscript, and revised the manuscript for important intellectual content.

DCE participated in the concept and design of the study, data acquisition, and interpretation, drafted the manuscript, and revised the manuscript for important intellectual content. MCTD participated in the data interpretation, drafted the manuscript, and revised the manuscript for important intellectual content. JAR participated in the data interpretation, drafted the manuscript, and revised the manuscript for important intellectual content. All authors read and approved the final version of the manuscript.

## Supplementary Material

Additional file 1 **Detailed search methods for identification of studies**. Click here for file

Additional file 2 **Table S1 presenting the assessment of study qualities**. Table S2 presenting the change in hemodynamic variables ((final - baseline value/baseline value) × 100%). Table S3 presenting the change in biochemical variables ((final - baseline value/baseline value) × 100%). Table S4 presenting adverse events. Table S5 presenting stratified analyses of pooled relative risks and standardized mean difference. Table S6 presenting Grading of Recommendations Assessment, Development and Evaluation (GRADE) evidence profile for impact of vasopressin or terlipressin for vasodilatory shock from systematic review and meta-analysis of randomized controlled trials. Click here for file

Additional file 3 **Figure S1 showing the standardized mean difference of the cardiac index**. Figure S2 showing the standardized mean difference of the oxygen delivery index (DO_2_i). Figure S3 showing the standardized mean difference of the oxygen consumption index (VO_2_i). Figure S4 showing the standardized mean difference of arterial lactate. Figure S5 showing the standardized mean difference of the gastric PaCO_2 _gap (Pr-aCO_2_). Figure S6 showing risk for adverse events. Click here for file

## References

[B1] Landry DW Oliver JA The pathogenesis of vasodilatory shock N Engl J Med 2001 345 588 595 10.1056/NEJMra002709 11529214

[B2] Dellinger RP Levy MM Carlet JM Bion J Parker MM Jaeschke R Reinhart K Angus DC Brun-Buisson C Beale R Calandra T Dhainaut JF Gerlach H Harvey M Marini JJ Marshall J Ranieri M Ramsay G Sevransky J Thompson BT Townsend S Vender JS Zimmerman JL Vincent JL International Surviving Sepsis Campaign Guidelines Committee; American Association of Critical-Care Nurses; American College of Chest Physicians; American College of Emergency Physicians; Canadian Critical Care Society; European Society of Clinical Microbiology and Infectious Diseases Surviving Sepsis Campaign: international guidelines for management of severe sepsis and septic shock: 2008 Crit Care Med 2008 36 296 327 10.1097/01.CCM.0000298158.12101.41 18158437

[B3] Mullner M Urbanek B Havel C Losert H Waechter F Gamper G Vasopressors for shock Cochrane Database Syst Rev 2004 3 CD003709 15266497 10.1002/14651858.CD003709.pub2

[B4] Holmes CL Walley KR Vasopressin in the ICU Curr Opin Crit Care 2004 10 442 448 10.1097/01.ccx.0000144769.19213.0c 15616384

[B5] Sutton AJ Higgins JP Recent developments in meta-analysis Stat Med 2008 27 625 650 10.1002/sim.2934 17590884

[B6] Albanèse J Leone M Delmas A Martin C Terlipressin or norepinephrine in hyperdynamic septic shock: a prospective, randomized study Crit Care Med 2005 33 1897 1902 10.1097/01.CCM.0000178182.37639.D6 16148457

[B7] Dünser MW Mayr AJ Ulmer H Knotzer H Sumann G Pajk W Friesenecker B Hasibeder WR Arginine vasopressin in advanced vasodilatory shock. A prospective, randomized, controlled study Circulation 2003 107 2313 2319 10.1161/01.CIR.0000066692.71008.BB 12732600

[B8] Morelli A Ertmer C Rehberg S Lange M Orecchioni A Cecchini V Bachetoni A D'Alessandro M Van Aken H Pietropaoli P Westphal M Continuous terlipressin versus vasopressin infusion in septic shock (TERLIVAP): a randomized, controlled pilot study Crit Care 2009 13 R130 R143 10.1186/cc7990 19664253 PMC2750187

[B9] Morelli A Ertmer C Lange M Dünser M Rehberg S Van Aken H Pietropaoli P Westphal M Effects of short-term simultaneous infusion of dobutamine and terlipressin in patients with septic shock: the DOBUPRESS study Br J Anaesth 2008 100 494 503 10.1093/bja/aen017 18308741

[B10] Russell JA Walley KR Singer J Gordon AC Hébert PC Cooper DJ Holmes CL Mehta S Granton JT Storms MM Cook DJ Presneill JJ Ayers D VASST Investigators Vasopressin versus norepinephrine infusion in patients with septic shock N Engl J Med 2008 358 877 887 10.1056/NEJMoa067373 18305265

[B11] Argenziano M Choudhri AF Oz MC Rose EA Smith CR Landry DW A prospective randomized trial of arginine vasopressin in the treatment of vasodilatory shock after left ventricular assist device placement Circulation 1997 96 286 290 9386112

[B12] Patel BM Chittock DR Russell JA Walley KR Beneficial effects of short-term vasopressin infusion during severe septic shock Anesthesiology 2002 96 576 582 10.1097/00000542-200203000-00011 11873030

[B13] Malay MB Ashton RC Landry DW Townsend RN Low-dose vasopressin in the treatment of vasodilatory septic shock J Trauma 1999 47 699 703 10.1097/00005373-199910000-00014 10528604

[B14] Lauzier F Lévy B Lamarre B Lesur O Vasopressin or norepinephrine in early hyperdynamic septic shock: a randomized clinical trial Intensive Care Med 2006 32 1782 1789 10.1007/s00134-006-0378-0 17019548

[B15] Barrett LK Singer M Clapp LH Vasopressin: mechanisms of action on the vasculature in health and in septic shock Crit Care Med 2007 35 33 40 10.1097/01.CCM.0000251127.45385.CD 17133186

[B16] Russell JA Vasopressin in vasodilatory and septic shock Curr Opin Crit Care 2007 13 383 391 10.1097/MCC.0b013e328263885e 17599007

[B17] Landry DW Levin HR Gallant EM Seo S D'Alessandro D Oz MC Oliver JA Vasopressin pressor hypersensitivity in vasodilatory septic shock Crit Care Med 1997 25 1279 1282 10.1097/00003246-199708000-00012 9267938

[B18] Avontuur JA Tutein Nolthenius RP Buijk SL Kanhai KJ Bruining HA Effect of L-NAME, an inhibitor of nitric oxide synthesis, on cardiopulmonary function in human septic shock Chest 1998 113 1640 1646 10.1378/chest.113.6.1640 9631805

[B19] Cobb JP Use of nitric oxide synthase inhibitors to treat septic shock: the light has changed from yellow to red Crit Care Med 1999 27 855 856 10.1097/00003246-199905000-00002 10362394

[B20] Klinzing S Simon M Reinhart K Bredle DL Meier-Hellmann A High-dose vasopressin is not superior to norepinephrine in septic shock Crit Care Med 2003 31 2646 2650 10.1097/01.CCM.0000094260.05266.F4 14605537

[B21] Singer M Matthay MA Clinical review: Thinking outside the box - an iconoclastic view of current practice Crit Care 2011 15 225 234 10.1186/cc10245 21888690 PMC3387582

[B22] Richard C Stress-related cardiomyopathies Ann Intensive Care 2011 1 39 46 10.1186/2110-5820-1-39 21933374 PMC3224539

[B23] Landesberg G Gilon D Meroz Y Georgieva M Levin PD Goodman S Avidan A Beeri R Weissman C Jaffe AS Sprung CL Diastolic dysfunction and mortality in severe sepsis and septic shock Eur Heart J 2012 33 895 903 10.1093/eurheartj/ehr351 21911341 PMC3345552

[B24] Lange M Ertmer C Rehberg S Morelli A Köhler G Kampmeier TG Van Aken H Westphal M Effects of two different dosing regimens of terlipressin on organ functions in ovine endotoxemia Inflamm Res 2011 60 429 437 10.1007/s00011-010-0299-9 21190124

[B25] Leone M Boyle WA Decreased vasopressin responsiveness in vasodilatory septic shock-like conditions Crit Care Med 2006 34 1126 1130 10.1097/01.CCM.0000206466.56669.BE 16484914

[B26] Dünser MW Hasibeder WR Wenzel V Vasopressin in septic shock N Engl J Med 2008 358 2736 18572442

[B27] Polito A Parisini E Ricci Z Picardo S Annane D Vasopressin for treatment of vasodilatory shock: an ESICM systematic review and meta-analysis Intensive Care Med 2012 38 9 19 10.1007/s00134-011-2407-x 22127480

