# Supplementary material for: Vasopressin and terlipressin in adult vasodilatory shock: a systematic review and meta-analysis of nine randomized controlled trials
Source: Crit Care. 2012 Aug 14;16(4):R154. doi: 10.1186/cc11469 (PMC3580743; doi:10.1186/cc11469)
Supplement: Additional file 1 — Detailed search methods for identification of studies. [file cc11469-S1.DOC]

**VASOPRESSIN AND TERLIPRESSIN IN ADULT VASODILATORY SHOCK: A systematic review and meta-analysis of nine randomized controlled trials**

ONLINE DATA SUPPLEMENT

Ary Serpa Neto, MD, MSc; Antônio P Nassar Júnior, MD; Sérgio O Cardoso, MD; José A Manetta, MD; Victor GM Pereira, MD; Daniel C Espósito, MD; Maria CT Damasceno, MD, PhD; James A. Russell, MD

**ADDITIONAL FILE 1**

**MATERIALS AND METHODS**

Search methods for identification of studies

Sensitivity search strategy using text words (tw) and Medical Subject Heading (mh):

*1) Population:*

shock [MeSH Terms] OR sepsis [MeSH Terms] OR multiple organ failure [MeSH Terms] OR “vasodilatory shock” [Text Word] OR “septic shock” [Text Word]

*2) Intervention:*

vasopressins [MeSH Terms] OR terlipressin [MeSH Terms] OR arginine vasopressin [MeSH Terms] OR “vasopressin” [Text Word]

*3) Study design:*

randomized controlled trial [MeSH Terms] OR clinical trial [MeSH Terms] OR controlled clinical trial [MeSH Terms]

*COMBINED:*

1 AND 2 AND 3
