# Supplementary material for: Vasopressin and terlipressin in adult vasodilatory shock: a systematic review and meta-analysis of nine randomized controlled trials
Source: Crit Care. 2012 Aug 14;16(4):R154. doi: 10.1186/cc11469 (PMC3580743; doi:10.1186/cc11469)
Supplement: Additional file 2 — Table S1 presenting the assessment of study qualities. Table S2 presenting the change in hemodynamic variables ((final - baseline value/baseline value) × 100%). Table S3 presenting the change in biochemical variables ((final - baseline value/baseline value) × 100%). Table S4 presenting adverse events. Table S5 presenting stratified analyses of pooled relative risks and standardized mean difference. Table S6 presenting Grading of Recommendations Assessment, Development and Evaluation (GRADE) evidence profile for impact of vasopressin or terlipressin for vasodilatory shock from systematic review and meta-analysis of randomized controlled trials. [file cc11469-S2.DOC]

**VASOPRESSIN AND TERLIPRESSIN IN ADULT VASODILATORY SHOCK: A systematic review and meta-analysis of nine randomized controlled trials**

ONLINE DATA SUPPLEMENT

Ary Serpa Neto, MD, MSc; Antônio P Nassar Júnior, MD; Sérgio O Cardoso, MD; José A Manetta, MD; Victor GM Pereira, MD; Daniel C Espósito, MD; Maria CT Damasceno, MD, PhD; James A. Russell, MD

ADDITIONAL FILE 2

| **Table S1 –** Assessment of study quality | | | | | | |
| --- | --- | --- | --- | --- | --- | --- |
| **Studies** | **Allocation Concealment** | **Blinding** | **Intention-to-treat Analysis** | **Lost to Follow-up** | **Early Stopping** | **Baseline similarity** |
| Albanese J, 2006 | Yes (Computer generated) | No | No | No | No | Yes |
| Dunser MW, 2002 | Yes (Random number) | No | No | 2% | No | Yes |
| Morelli A, 2009 | Yes (Computer generated) | No | No | No | No | Yes |
| Morelli A, 2008 | Yes (Computer generated) | No | No | 1.6% | No | Yes |
| Russell JA, 2008 | Yes (Computer generated) | Double | No | 3% | No | No (age and number of organ dysfunctions) |
| Argenziano M, 1997 | Unclear | Double | No | No | No | Yes |
| Patel BM, 2002 | Yes (Computer generated) | Double | No | No | No | Yes |
| Malay MB, 1999 | Yes (Computer generated) | Double | No | No | No | Yes |
| Lauzier F, 2006 | Yes (Computer generated) | No | Yes | 4.3% | No | No (bilirubin) |

**ADDITIONAL FILE 2**

| **Table S2 –** Change in hemodynamic variables ([final – baseline value / baseline value] x 100%) | | | | | | | | | | | | | | | |
| --- | --- | --- | --- | --- | --- | --- | --- | --- | --- | --- | --- | --- | --- | --- | --- |
| **Study** | **Arms** | **HR** | **MAP** | **Ci** | **SVi** | **PAOP** | **SVRi** | **MPAP** | **PVRi** | **DO_2_i** | **LVSWi** | **RVSWi** | **VO_2_i** | **CVP** | **O_2_ER** |
| Albanese J, 2006 | N  TP | 4  -16 | 33  27 | 4  -20 | 5  -2 | 0  -7 | 44  97 | 17  17 | 29  79 | 1  -14 | 56  64 | 150  67 | -4  -12 | --- | -7  -3 |
| Dunser MW, 2002 | N  N+AV | -5  -19 | 12  28 | 3  0 | 0  22 | -6  0 | 11  15 | -14  -3 | --- | 4  8 | 25  69 | --- | 8  -2 | --- | 4  -3 |
| Morelli A, 2009 | N  N+TP  N+AV | -1  -25  -7 | 31  34  34 | -2  -12  5 | -2  9  15 | 7  13  13 | 51  52  46 | 25  12  21 | 63  33  32 | 1  -15  12 | 41  68  62 | 28  25  22 | 0  -17  0 | 17  17  18 | -3  0  -5 |
| Morelli A, 2008 | N  N+TP | 2  -12 | 1  5 | -10  -17 | --- | 0  7 | 4  29 | 0  4 | 2  24 | -4  -19 | -6  -3 | -12  0 | -2  -5 | 0  -8 | 6  21 |
| Russell JA, 2008 | N  N+AV | -6  -7 | 11  11 | --- | --- | --- | --- | --- | --- | --- | --- | --- | --- | --- | --- |
| Argenziano M, 1997 | N+P  N+AV | --- | 3  47 | 2  5 | --- | --- | 2  46 | --  -8 | --- | --- | --- | --- | --- | --- | --- |
| Patel BM, 2002 | N  N+AV | -5  -9 | -1  0 | -20  -8 | --- | 21  33 | 4  5 | --- | -12  4 | --- | --- | --- | --- | --- | --- |
| Malay MB, 1999 | N+P  N+AV | 10  -14 | 21  26 | -25  -7 | --- | 5  25 | 71  54 | --- | --- | --- | --- | --- | --- | --- | --- |
| Lauzier F, 2006 | N  N+AV | -12  -21 | 19  8 | -16  -19 | 1  5 | 21  8 | 42  52 | 13  -10 | 36  3 | -12  -27 | --- | --- | -7  -13 | 15  20 | 5  19 |
| **Total (mean)** | C  AV  TP | -1.62  -12.8  -17.3 | 14.4  22.0  22.0 | -5.62  -4.00  -16.3 | 1.00  14.0  3.50 | 6.85  15.8  4.33 | 28.6  36.3  59.3 | 8.20  -2.25  11.0 | 23.6  13.0  45.3 | -2.00  -2.33  -16.0 | 29.0  65.0  43.0 | 55.3  22.0  30.6 | -1.00  -5.00  -11.3 | 10.6  19.0  4.50 | 1.00  3.66  6.00 |
| N: Norepinephrine; TP: Terlipressin; P: Placebo; D: Drug that the patients were already receiving at baseline; AV: Arginine Vasopressin; C: Control  HR: Heart rate; MAP: Mean arterial pressure; Ci: Cardiac index; SVi: Stroke volume index; PAOP: Pulmonary artery occlusion pressure; SVRi: Systemic vascular resistance index; MPAP: Mean pulmonary artery pressure; PVRi: Pulmonary vascular resistance index; DO_2_i: Systemic oxygen transport index; LVSWi: Left ventricular stroke work index; RVSWI: Right ventricular stroke work index; VO_2_i: Systemic oxygen consumption index; CVP: Central venous pressure; O_2_ER: Systemic oxygen extraction rate. | | | | | | | | | | | | | | | |

**ADDITIONAL FILE 2**

| **Table S3 –** Change in biochemical variables ([final – baseline value / baseline value] x 100%) | | | | | | | | | | | | |
| --- | --- | --- | --- | --- | --- | --- | --- | --- | --- | --- | --- | --- |
| **Study** | **Arms** | **pH** | **PaCO_2_** | **Lactate** | **Creatinine** | **AST** | **ALT** | **Bilirrubin** | **Platelets** | **Pr-aCO_2_** | **Troponin** | **INR** |
| Albanese J, 2006 | N  TP | 0  0 | -3  -3 | -47  -37 | --- | --- | --- | --- | --- | --- | --- | --- |
| Dunser MW, 2002 | N  N+AV | 1  1 | 1  0 | -55  -58 | 3  -4 | -65  -56 | -43  -18 | 34  99 | -6  -55 | 75  122 | -53  -60 | --- |
| Morelli A, 2009 | N  N+TP  N+AV | -1  1  0 | -2  -15  -5 | 39  16  13 | 50  12  10 | 25  -8  44 | 46  0  80 | 75  -25  44 | -17  -41  -15 | 29  -13  12 | 9  -42  109 | -7  0  0 |
| Morelli A, 2008 | N  N+TP | 0  1 | 2  2 | 23  -17 | --- | -5  -6 | 6  15 | 7  7 | --- | 7  0 | 62  112 | -1  -4 |
| Patel BM, 2002 | N  N+AV | --- | --- | --- | --- | --- | --- | --- | --- | -14  35 | --- | --- |
| Malay MB, 1999 | N+P  N+AV | --- | --- | --- | 31  9 | --- | --- | --- | --- | --- | --- | --- |
| Lauzier F, 2006 | N  N+AV | --- | --- | -13  -22 | --- | --- | --- | 9  -19 | -22  -30 | -20  14 | --- | --- |
| **Total (mean)** | C  AV  TP | 0.00  0.00  0.66 | -0.50  -5.00  -5.33 | -10.6  -4.50  -12.6 | 28.0  9.50  12.0 | -15.0  44.0  -7.0 | 3.00  80.0  7.5 | 39.6  19.6  -9.00 | -21.0  -1.33  -41.0 | 15.4  20.3  -6.50 | 6.00  109.0  35.0 | 1.66  2.00  -2.00 |
| N: Norepinephrine; TP: Terlipressin; P: Placebo; D: Drug that the patients were already receiving at baseline; AV: Arginine Vasopressin; C: Control  AST: Aspartate aminotransferase; ALT: Alanine aminotransferase; Pr-aCO_2_: Gastric-arterial PCO_2_ difference; INR: International normalized ratio | | | | | | | | | | | | |

**ADDITIONAL FILE 2**

| **Table S4 –** Adverse events | | | | | | | |
| --- | --- | --- | --- | --- | --- | --- | --- |
| **Study** | **Arms** | **Total** | **Digital ischemia** | **Bradycardia** | **Cardiac Arrest** | **Tachyarrhythmias** | **MI** |
| Dunser MW, 2002 | N  N+AV | --- | 6  7 | 1  -- | --- | 14  2 | 2  1 |
| Morelli A, 2009 | N  N+TP  N+AV | --- | --- | --- | --- | 4  0  1 | --- |
| Russell JA, 2008 | N  N+AV | 40  41 | 2  8 | --- | 8  3 | 6  8 | 7  8 |
| Malay MB, 1999 | N+P  N+AV | 0  0 | 0  0 | --- | --- | --- | 0  0 |
| Lauzier F, 2006 | N  N+AV | 1  1 | 0  0 | --- | --- | 0  0 | 1  1 |
| **Total (sum)** | C  AV  TP | 41  42  --- | 11  15  5 | 1  0  --- | 8  3  --- | 24  11  0 | 10  10  --- |
| N: Norepinephrine; TP: Terlipressin; P: Placebo; D: Drug that the patients were already receiving at baseline; AV: Arginine Vasopressin; C: Control  MI: Myocardial infarction. | | | | | | | |

**ADDITIONAL FILE 2**

| **Table S5 –** Stratified analyses of pooled relative risks and standardized mean difference | | | | | | | | |
| --- | --- | --- | --- | --- | --- | --- | --- | --- |
| **Stratified analysis** | **Trials** | **N** | **Vasopressin** | ***p*** | **Heterogeneity** | **Terlipressin** | ***p*** | **Heterogeneity** |
| **Mortality** | | | | | | | | |
| Design  Double-blind  Open-label | 3  3 | 799  101 | 0.87 (0.75 – 1.01)  0.91 (0.66 – 1.24) | 0.07  0.55 | 0.48  0.78 | ---  0.88 (0.62 – 1.25) | ---  0.47 | ---  0.61 |
| Disease  Septic shock  Non-septic shock | 4  2 | 989  58 | 0.87 (0.75 – 1.00)  0.95 (0.65 – 1.37) | 0.05  0.77 | 0.76  0.46 | 0.88 (0.62 – 1.25)  --- | 0.47  --- | 0.61  --- |
| Vasopressin dosage  ≤ 0.05 U/min  > 0.05 U/min | 3  3 | 819  81 | 0.87 (0.75 – 1.01)  0.92 (0.63 – 1.33) | 0.06  0.66 | 0.56  0.70 | ---  --- | ---  --- | ---  --- |
| Terlipressin dosage  ≤ 40 µg/h  > 40 µg/h | 1  2 | 30  50 | ---  --- | ---  --- | ---  --- | 0.70 (0.37 – 1.34)  0.86 (0.50 – 1.47) | 0.28  0.57 | ---  0.33 |
| Follow-up  ≤ 24 hours  > 24 hours | 4  6 | 79  910 | 0.25 (0.03 – 1.92)  0.88 (0.77 – 1.01) | 0.18  0.08 | 0.81  0.90 | 0.98 (0.65 – 1.49)  0.70 (0.37 – 1.34) | 0.93  0.28 | 0.54  --- |
| Patients  ≤ 25  25 – 50  > 50 | 4  4  1 | 63  147  779 | 0.49 (0.16 – 1.49)  0.93 (0.68 – 1.27)  0.88 (0.76 – 1.02) | 0.21  0.63  0.10 | 0.65  0.52  --- | 1.25 (0.47 – 3.33)  0.82 (0.69 – 1.11)  --- | 0.66  0.29  --- | ---  0.52  --- |
| VASST vs. Others  VASST  Others | 1  5 | 779  121 | 0.88 (0.76 – 1.02)  0.83 (0.61 – 1.14) | 0.10  0.26 | ---  0.67 | ---  --- | ---  --- | ---  --- |
| In Septic Shock  VASST  Others | 1  3 | 779  63 | 0.88 (0.76 – 1.02)  0.70 (0.40 – 1.22) | 0.10  0.21 | ---  0.61 | ---  --- | ---  --- | ---  --- |
| Terlipressin infusion  *Bolus*  Continuous | 2  1 | 59  30 | ---  --- | ---  --- | ---  --- | 0.98 (0.65 – 1.49)  0.70 (0.37 – 1.34) | 0.93  0.28 | 0.54  --- |
| **Norepinephrine reduction** | | | | | | | | |
| Design  Double-blind  Open-label | 3  5 | 813  170 | -1.75 (-1.91 – -1.59)  -0.45 (-0.85 – -0.06) | < 0.0001  0.03 | < 0.0001  0.70 | ---  -1.97 (-2.62 – -1.32) | ---  < 0.0001 | ---  < 0.0001 |
| Disease  Septic shock  Non-septic shock | 6  2 | 925  48 | -1.64 (-1.79 – -1.48)  -0.66 (-1.22 – -0.11) | < 0.0001  0.02 | < 0.0001  0.03 | -1.97 (-2.62 – -1.32)  --- | < 0.0001  --- | < 0.0001  --- |
| Vasopressin dosage  ≤ 0.05 U/min  > 0.05 U/min | 2  4 | 809  105 | -1.66 (-1.83 – -1.50)  -0.77 (-1.22 – -0.32) | < 0.0001  0.0008 | 0.005  < 0.0001 | ---  --- | ---  --- | ---  --- |
| Terlipressin dosage  ≤ 40 µg/h  > 40 µg/h | 1  1 | 39  30 | ---  --- | ---  --- | ---  --- | -4.63 (-5.88 – -3.38)  -0.98 (-1.74 – -0.22) | < 0.0001  0.01 | ---  --- |
| Follow-up  ≤ 24 hours  > 24 hours | 3  5 | 73  910 | -4.62 (-6.11 – -3.13)  -1.53 (-1.68 – -1.38) | < 0.0001  < 0.0001 | 0.02  < 0.0001 | -4.63 (-5.88 – -3.38)  -0.98 (-1.74 – -0.22) | < 0.0001  0.01 | ---  --- |
| Patients  ≤ 25  25 – 50  > 50 | 3  4  1 | 57  147  779 | -1.21 (-1.93 – -0.49)  -0.54 (-1.00 – -0.09)  -1.72 (-1.88 – -1.55) | 0.001  0.02  < 0.0001 | < 0.0001  0.75  --- | ---  -1.97 (-2.62 – -1.32)  --- | ---  < 0.0001  --- | ---  < 0.0001  --- |
| VASST vs. Others  VASST  Others | 1  5 | 779  135 | -1.72 (-1.88 – -1.55)  -0.73 (-1.11 – -0.35) | < 0.0001  0.0002 | ---  < 0.0001 | ---  --- | ---  --- | ---  --- |
| Terlipressin infusion  *Bolus*  Continuous | 1  1 | 59  30 | ---  --- | ---  --- | ---  --- | -4.63 (-5.88 – -3.38)  -0.98 (-1.74 – -0.22) | <0.0001  0.01 | ---  --- |
| **Cardiac Index** | | | | | | | | |
| Design  Double-blind  Open-label | 3  6 | 46  190 | 0.94 (0.29 – 1.58)  0.03 (-0.36 – 0.42) | 0.004  0.89 | 0.45  0.80 | ---  -0.44 (-0.87 – -0.02) | ---  0.04 | ---  0.54 |
| Disease  Septic shock  Non-septic shock | 7  2 | 176  58 | 0.44 (-0.00 – 0.88)  0.05 (-0.47 – 0.56) | 0.05  0.86 | 0.10  0.70 | -0.44 (-0.87 – -0.02)  --- | 0.04  --- | 0.54  --- |
| Vasopressin dosage  ≤ 0.05 U/min  > 0.05 U/min | 2  4 | 40  105 | 0.44 (-0.20 – 1.09)  0.21 (-0.18 – 0.60) | 0.18  0.29 | 0.15  0.15 | ---  --- | ---  --- | ---  --- |
| Terlipressin dosage  ≤ 40 µg/h  > 40 µg/h | 1  2 | 39  50 | ---  --- | ---  --- | ---  --- | -0.29 (-0.92 – 0.35)  -0.57 (-1.14 – -0.00) | 0.38  0.05 | ---  0.37 |
| Follow-up  ≤ 24 hours  > 24 hours | 5  4 | 103  131 | 0.94 (0.29 – 1.58)  0.03 (-0.36 – 0.42) | 0.004  0.89 | 0.45  0.80 | -0.48 (-1.00 – 0.04)  -0.37 (-1.09 – 0.35) | 0.07  0.31 | 0.28  --- |
| Patients  ≤ 25  25 – 50 | 5  4 | 87  147 | 0.52 (0.02 – 1.03)  0.08 (-0.36 – 0.53) | 0.04  0.72 | 0.12  0.65 | -0.91 (-1.84 – 0.02)  -0.32 (-0.80 – 0.15) | 0.06  0.18 | ---  0.86 |
| Terlipressin infusion  *Bolus*  Continuous | 2  1 | 59  30 | ---  --- | ---  --- | ---  --- | -0.48 (-1.00 – 0.04)  -0.37 (-1.09 – 0.35) | 0.07  0.31 | 0.28  --- |
| **DO_2_** | | | | | | | | |
| Disease  Septic shock  Non-septic shock | 5  1 | 142  48 | -0.06 (-0.61 – 0.48)  -0.07 (-0.64 – 0.49) | 0.82  0.80 | 0.15  --- | -0.79 (-1.23 – -0.36)  --- | 0.0004  --- | 0.66  --- |
| Vasopressin dosage  ≤ 0.05 U/min  > 0.05 U/min | 1  2 | 30  71 | 0.28 (-0.44 – 1.00)  -0.22 (-0.69 – 0.25) | 0.44  0.36 | ---  0.37 | ---  --- | ---  --- | ---  --- |
| Terlipressin dosage  ≤ 40 µg/h  > 40 µg/h | 1  2 | 39  50 | ---  --- | ---  --- | ---  --- | -0.75 (-1.40 – -0.10)  -0.83 (-1.42 – -0.25) | 0.02  0.005 | ---  0.38 |
| Follow-up  ≤ 24 hours  > 24 hours | 2  4 | 59  131 | ---  -0.07 (-0.46 – 0.32) | ---  0.73 | ---  0.35 | -0.88 (-1.42 – -0.34)  -0.63 (-1.36 – 0.11) | 0.001  0.09 | 0.47  --- |
| Patients  ≤ 25  25 – 50 | 2  4 | 43  147 | -0.54 (-1.38 – 0.30)  0.06 (-0.38 – 0.51) | 0.21  0.78 | ---  0.45 | -1.18 (-2.15 – -0.21)  -0.70 (-1.18 – -0.21) | 0.02  0.005 | ---  0.81 |
| Terlipressin infusion  *Bolus*  Continuous | 2  1 | 59  30 | ---  --- | ---  --- | ---  --- | -0.88 (-1.42 – -0.34)  -0.63 (-1.36 – 0.11) | 0.001  0.09 | 0.47  --- |
| **VO_2_** | | | | | | | | |
| Disease  Septic shock  Non-septic shock | 5  1 | 142  48 | -0.09 (-0.63 – 0.45)  -0.29 (-0.86 – 0.28) | 0.75  0.32 | 0.76  --- | -0.34 (-0.76 – 0.08)  --- | 0.11  --- | 0.60  --- |
| Vasopressin dosage  ≤ 0.05 U/min  > 0.05 U/min | 1  2 | 30  71 | -0.02 (-0.73 – 0.70)  -0.26 (-0.72 – 0.21) | 0.96  0.29 | ---  0.84 | ---  --- | ---  --- | ---  --- |
| Terlipressin dosage  ≤ 40 µg/h  > 40 µg/h | 1  2 | 39  50 | ---  --- | ---  --- | ---  --- | -0.13 (-0.76 – 0.50)  -0.52 (-1.08 – 0.05) | 0.69  0.07 | ---  0.64 |
| Follow-up  ≤ 24 hours  > 24 hours | 2  4 | 59  131 | ---  -0.18 (-0.58 – 0.21) | ---  0.36 | ---  0.84 | -0.21 (-0.72 – 0.31)  -0.63 (-1.36 – 0.11) | 0.43  0.09 | 0.68  --- |
| Patients  ≤ 25  25 – 50 | 2  4 | 43  147 | -0.19 (-1.01 – 0.64)  -0.18 (-0.63 – 0.26) | 0.66  0.42 | ---  0.56 | -0.36 (-1.24 – 0.53)  -0.34 (-0.82 – 0.14) | 0.43  0.16 | ---  0.31 |
| Terlipressin infusion  *Bolus*  Continuous | 2  1 | 59  30 | ---  --- | ---  --- | ---  --- | -0.21 (-0.72 – 0.31)  -0.63 (-1.36 – 0.11) | 0.43  0.09 | 0.68  --- |
| **Arterial Lactate** | | | | | | | | |
| Design  Double-blind  Open-label | 1  6 | 55  190 | 0.00 (-0.49 – 0.49)  -0.18 (-0.57 – 0.22) | 1.00  0.38 | ---  0.95 | ---  -0.32 (-0.75 – 0.10) | ---  0.13 | ---  0.22 |
| Disease  Septic shock  Non-septic shock | 5  2 | 142  113 | -0.22 (-0.77 – 0.32)  -0.05 (-0.42 – 0.32) | 0.42  0.78 | 0.83  0.75 | -0.32 (-0.75 – 0.10)  --- | 0.13  --- | 0.22  --- |
| Vasopressin dosage  ≤ 0.05 U/min  > 0.05 U/min | 2  5 | 95  71 | -0.09 (-0.49 – 0.32)  -0.13 (-0.60 – 0.33) | 0.67  0.58 | 0.53  0.95 | ---  --- | ---  --- | ---  --- |
| Terlipressin dosage  ≤ 40 µg/h  > 40 µg/h | 1  2 | 39  50 | ---  --- | ---  --- | ---  --- | -0.70 (-1.35 – -0.05)  -0.05 (-0.60 – 0.51) | 0.03  0.87 | ---  0.36 |
| Follow-up  ≤ 24 hours  > 24 hours | 3  4 | 114  131 | 0.00 (-0.49 – 0.49)  -0.18 (-0.57 – 0.22) | 1.00  0.38 | ---  0.95 | -0.36 (-0.88 – 0.17)  -0.26 (-0.98 – 0.46) | 0.18  0.48 | 0.08  --- |
| Patients  ≤ 25  25 – 50  > 50 | 2  4  1 | 43  147  55 | -0.16 (-0.98 – 0.67)  -0.18 (-0.63 – 0.26)  0.00 (-0.49 – 0.49) | 0.71  0.42  1.00 | ---  0.74  --- | 0.27 (-0.61 – 1.15)  -0.50 (-0.98 – -0.02)  --- | 0.54  0.04  --- | ---  0.38  --- |
| **Pr-aCO_2_** | | | | | | | | |
| Design  Double-blind  Open-label | 1  5 | 24  170 | 1.29 (0.39 – 2.19)  0.02 (-0.37 – 0.42) | 0.005  0.91 | ---  0.36 | ---  -0.47 (-0.96 – 0.01) | ---  0.06 | ---  0.11 |
| Disease  Septic shock  Non-septic shock | 5  1 | 146  48 | 0.31 (-0.15 – 0.78)  0.10 (-0.47 – 0.66) | 0.19  0.73 | 0.02  --- | -0.47 (-0.40 – 0.27)  --- | 0.71  --- | 0.003  --- |
| Vasopressin dosage  ≤ 0.05 U/min  > 0.05 U/min | 1  3 | 30  95 | -0.38 (-1.11 – 0.34)  0.43 (0.01 – 0.84) | 0.30  0.04 | ---  0.09 | ---  --- | ---  --- | ---  --- |
| Terlipressin dosage  ≤ 40 µg/h  > 40 µg/h | 1  1 | 39  30 | ---  --- | ---  --- | ---  --- | -0.15 (-0.78 – 0.48)  -0.94 (-1.70 – -0.18) | 0.64  0.02 | ---  --- |
| Follow-up  ≤ 24 hours  > 24 hours | 2  4 | 63  131 | 1.29 (0.39 – 2.19)  0.02 (-0.37 – 0.42) | 0.005  0.91 | ---  0.36 | -0.15 (-0.78 – 0.48)  -0.94 (-1.70 – -0.18) | 0.64  0.02 | ---  --- |
| Patients  ≤ 25  25 – 50 | 2  4 | 47  147 | 0.81 (0.20 – 1.42)  -0.09 (-0.53 – 0.36) | 0.009  0.71 | 0.15  0.30 | ---  -0.47 (-0.96 – 0.01) | ---  0.06 | ---  0.11 |
| Relative risks (IC 95%) for mortality and adverse events  Standardized mean difference (IC 95%) for cardiac index, norepinephrine reduction, DO_2_, VO_2_, arterial lactate and Pr-aCO_2_ | | | | | | | | |

**ADDITIONAL FILE 2**

| **Table S6 –** GRADE evidence profile for impact of vasopressin or terlipressin for vasodilatory shock from systematic review and meta-analysis of randomized controlled trials | | | | | | | | | |
| --- | --- | --- | --- | --- | --- | --- | --- | --- | --- |
|  | **Quality assessment** | | | | |  | **Summary of findings** | | |
| **No of studies**  **(No of participants)** | **Study limitations** | **Consistency** | **Directness** | **Precision** | **Publication bias** |  | **Relative effect**  **(95% CI)** | **Best estimate of intervention group** | **Quality** |
| **Vasopressin vs. Control (General)** | | | | | | | | | |
| Mortality: | | | | | | | | | |
| 6 (900) | Moderate limitations^*^ | No important inconsistency | Direct | Not important imprecision | Unlikely |  | 0.87 (0.76 to 1.00) | 0% | ++++, high |
| Norepinephrine reduction: | | | | | | | | | |
| 6 (914) | Serious limitations^**^ | Unexplained Heterogeneity | Direct | Not important imprecision | Unlikely |  | -1.56 (-1.71 to -1.41) | -6.45 | ++, low |
| Adverse events: | | | | | | | | | |
| 3 (812) | Low limitations^***^ | No important inconsistency | Direct | Imprecision | Unlikely |  | 0.98 (0.65 to 1.47) | 0% | +++, moderate |
| **Vasopressin vs. Control (Septic Shock)** | | | | | | | | | |
| Mortality: | | | | | | | | | |
| 4 (842) | Serious limitations^¶^ | No important inconsistency | Direct | Not important imprecision | Unlikely |  | 0.87 (0.75 to 1.00) | 0% | +++, moderate |
| **Terlipressin vs. Control (General)** | | | | | | | | | |
| Mortality: | | | | | | | | | |
| 3 (89) | Serious limitations^¶¶^ | No important inconsistency | Direct | Imprecision | Unlikely |  | 0.88 (0.62 to 1.25) | 46.6% | ++, low |
| Norepinephrine reduction: | | | | | | | | | |
| 2 (69) | Serious limitations^║^ | Unexplained Heterogeneity | Direct | Not important imprecision | Unlikely |  | -1.58 (-1.73 to -1.44) | -4.63 | ++, low |
| *Unclear allocation concealment in one study, patients not blinded in three studies, not analyzed using intention-to-treat in four studies  **Unclear allocation concealment in one study, patients not blinded in three studies, not analyzed using intention-to-treat in five studies  ***Unclear allocation concealment in none study, patients not blinded in one studies, not analyzed using intention-to-treat in two studies  ¶Unclear allocation concealment in none study, patients not blinded in two studies, not analyzed using intention-to-treat in three studies  ¶¶Unclear allocation concealment in none study, patients not blinded in all studies, not analyzed using intention-to-treat in all studies  ^║^ Unclear allocation concealment in none study, patients not blinded in all studies, not analyzed using intention-to-treat in all studies | | | | | | | | | |
