# Supplementary material for: Vasopressin and terlipressin in adult vasodilatory shock: a systematic review and meta-analysis of nine randomized controlled trials
Source: Crit Care. 2012 Aug 14;16(4):R154. doi: 10.1186/cc11469 (PMC3580743; doi:10.1186/cc11469)
Supplement: Additional file 3 — Figure S1 showing the standardized mean difference of the cardiac index. Figure S2 showing the standardized mean difference of the oxygen delivery index (DO2i). Figure S3 showing the standardized mean difference of the oxygen consumption index (VO2i). Figure S4 showing the standardized mean difference of arterial lactate. Figure S5 showing the standardized mean difference of the gastric PaCO2 gap (Pr-aCO2). Figure S6 showing risk for adverse events. [file cc11469-S3.DOC]

**VASOPRESSIN AND TERLIPRESSIN IN ADULT VASODILATORY SHOCK: A systematic review and meta-analysis of nine randomized controlled trials**

ONLINE DATA SUPPLEMENT

Ary Serpa Neto, MD, MSc; Antônio P Nassar Júnior, MD; Sérgio O Cardoso, MD; José A Manetta, MD; Victor GM Pereira, MD; Daniel C Espósito, MD; Maria CT Damasceno, MD, PhD; James A. Russell, MD

**ADDITIONAL FILE 3**

**Figure S1 – Standardized mean difference of cardiac index**


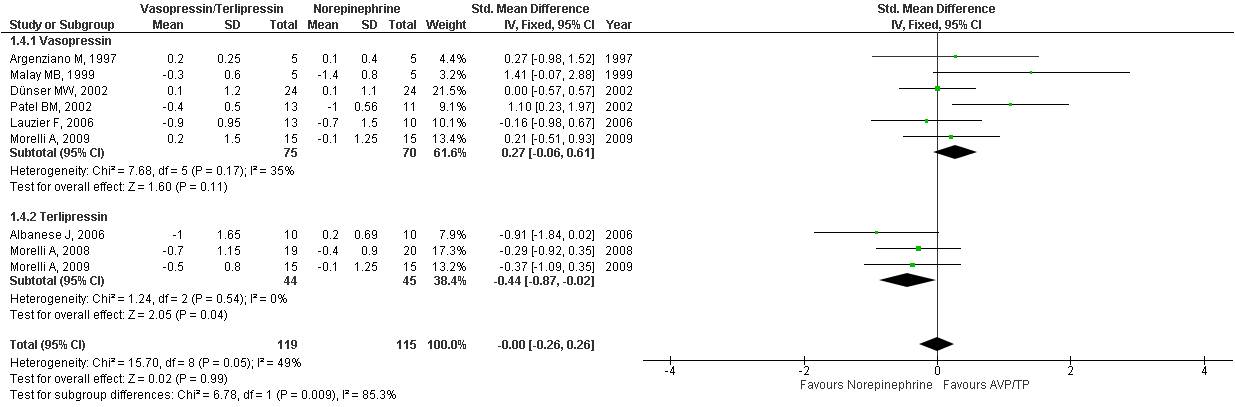


Standardized mean difference of cardiac index between AVP/TP and NE alone.

**ADDITIONAL FILE 3**

**Figure S2 – Standardized mean difference of DO2i**

**
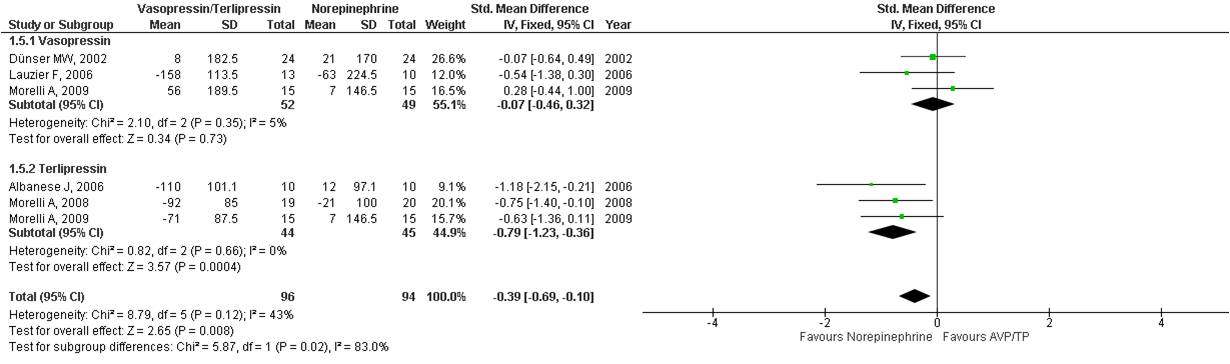
**

Standardized mean difference of DO2i between AVP/TP and NE alone.

**ADDITIONAL FILE 3**

**Figure S3 – Standardized mean difference of VO2i**

**
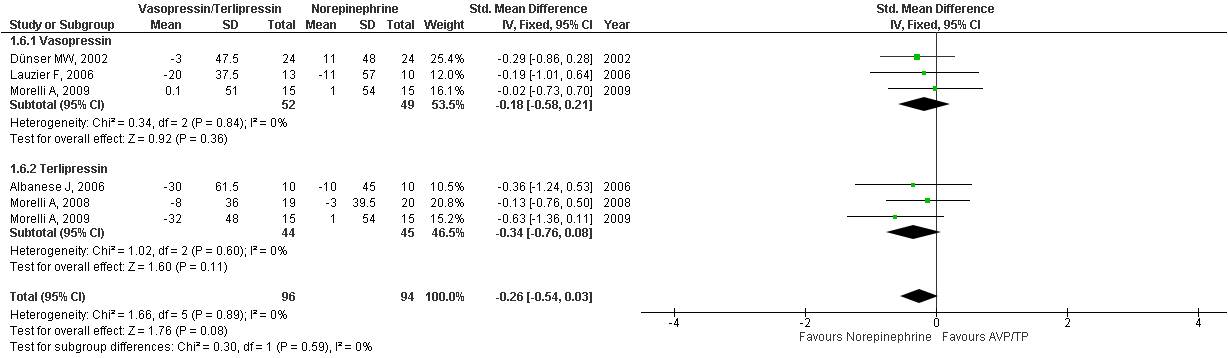
**

Standardized mean difference of VO2i between AVP/TP and NE alone.

**ADDITIONAL FILE 3**

**Figure S4 – Standardized mean difference of arterial lactate**

**
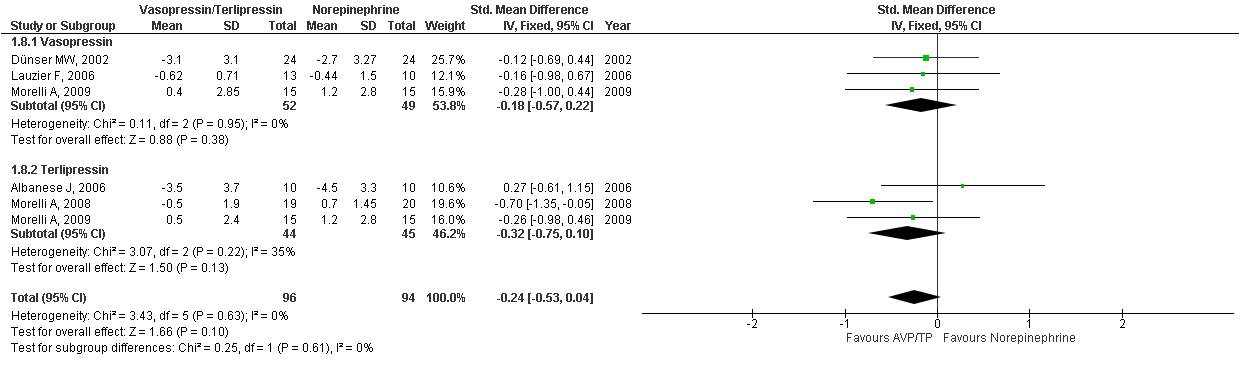
**

Standardized mean difference of arterial lactate between AVP/TP and NE alone.

**ADDITIONAL FILE 3**

**Figure S5 – Standardized mean difference of Pr-aCO2**

**
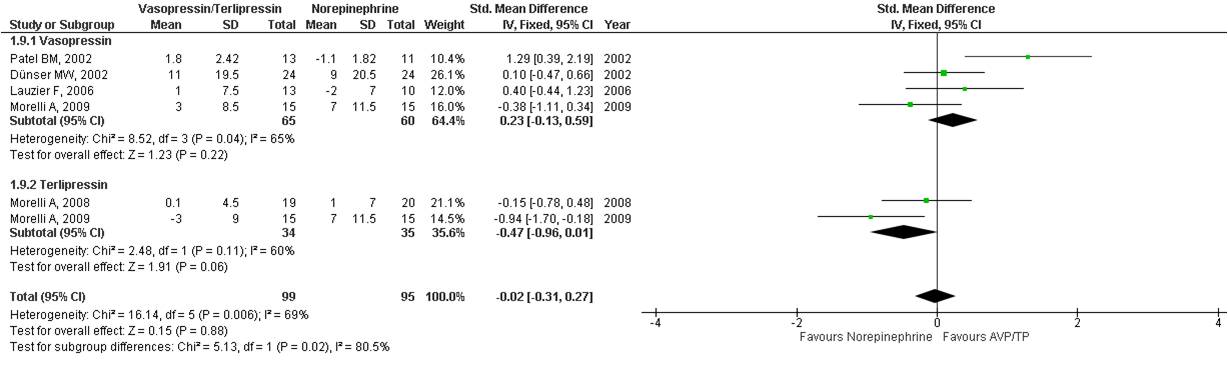
**

Standardized mean difference of Pr-aCO2 between AVP/TP and NE alone.

**ADDITIONAL FILE 3**

**Figure S6 – Risk for adverse events
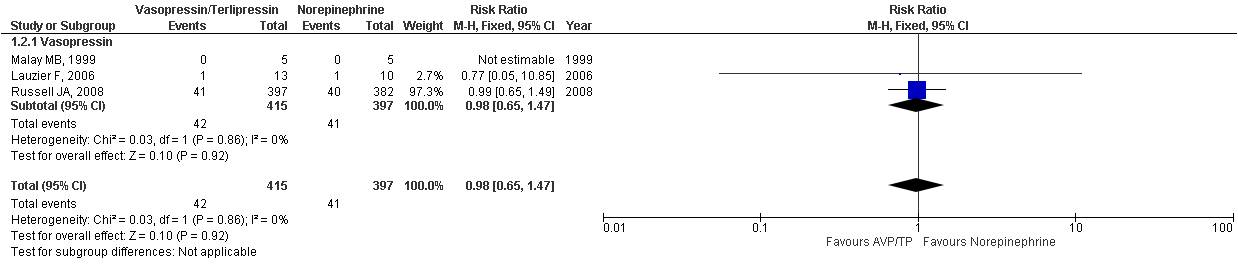
**

Risk for adverse events relative to NE alone for participants with vasodilatory shock under AVP infusion.
